# High density of FOXP3-positive T cells infiltrating colorectal cancers with microsatellite instability

**DOI:** 10.1038/sj.bjc.6604756

**Published:** 2008-11-04

**Authors:** S Michel, A Benner, M Tariverdian, N Wentzensen, P Hoefler, T Pommerencke, N Grabe, M von Knebel Doeberitz, M Kloor

**Affiliations:** 1 Molecular Medicine Partnership Unit (MMPU), Department of Applied Tumour Biology, Institute of Pathology, University of Heidelberg, Im Neuenheimer Feld 220/221, Heidelberg 69120, Germany; 2 Group Cancer Early Detection, German Cancer Research Center (DKFZ), Im Neuenheimer Feld 280, Heidelberg 69120, Germany; 3 Division of Biostatistics, German Cancer Research Center (DKFZ), Im Neuenheimer Feld 280, Heidelberg 69120, Germany; 4 Department of Surgery, University of Heidelberg, Im Neuenheimer Feld 110, Heidelberg 69120, Germany; 5 Hamamatsu TIGA Center (BIOQUANT), Institute of Medical Biometry and Informatics, University of Heidelberg, Im Neuenheimer Feld 305, Heidelberg 69120, Germany

**Keywords:** colorectal cancer, microsatellite instability, regulatory T cells, tumour immunity, immune evasion

## Abstract

High-level microsatellite instability (MSI-H) in colorectal cancer accounts for about 12% of colorectal cancers and is typically associated with a dense infiltration with cytotoxic CD8-positive lymphocytes. The role of regulatory T cells that may interfere with the host's antitumoural immune response in MSI-H colorectal cancers has not been analysed yet. Using an antibody directed against the regulatory T-cell marker transcription factor forkhead box P3 (FOXP3), regulatory T cells were examined in 70 colorectal cancers with known MSI status (MSI-H, *n*=37; microsatellite stable, *n*=33). In MSI-H colorectal cancers, we found a significantly higher intraepithelial infiltration with FOXP3-positive cells (median: 8.5 cells per 0.25 mm^2^ *vs* 3.1 cells per 0.25 mm^2^ in microsatellite stable, *P*<0.001), and a significantly elevated ratio of intraepithelial to stromal infiltration (0.05 *vs* 0.01 in microsatellite stable, *P*<0.001). CD8-positive cell counts were related positively to the number of FOXP3-positive cells (Spearman's *ρ*=0.56 and 0.55, respectively). Our results show that the elevated number of CD8-positive lymphocytes found in MSI-H colorectal cancers is paralleled by an enhanced infiltration with CD8-negative FOXP3-positive cells. These data suggest that FOXP3-positive cells may play a role in the regulation of the immune response directed against MSI-H colorectal cancers at the primary tumour site.

Colorectal cancers (CRCs) arise through two major molecular pathways. Although the majority of CRCs is hallmarked by large chromosomal alterations (Lengauer *et al*, 1997), about 10–15% of CRCs are characterised by the high-level microsatellite instability (MSI-H) phenotype that results from defects in the DNA mismatch repair system. The anti-tumoural immune response against CRCs is apparently closely linked to the molecular pathogenesis of these tumours, and MSI-H CRCs typically present with features of a pronounced local immune response, for example a high density of tumour-infiltrating lymphocytes with a high proportion of activated and cytotoxic CD8-positive lymphocytes (Dolcetti *et al*, 1999; Smyrk *et al*, 2001; Phillips *et al*, 2004; Jenkins *et al*, 2007). In line with the pronounced immunogenicity of MSI-H CRCs, these cancers rarely develop metastases in distant organs and have a comparably good prognosis (Buckowitz *et al*, 2005; Popat *et al*, 2005) in spite of a large tumour mass at the primary localisation (Wright *et al*, 2000).

The immunogenicity of MSI-H CRCs is ascribed to the generation of multiple immunogenic frameshift-derived antigens as a consequence of coding microsatellite mutations that result from mismatch repair deficiency (Linnebacher *et al*, 2001; Saeterdal *et al*, 2001; Schwitalle *et al*, 2008). The presence of these well-defined tumour-specific antigens renders MSI-H tumours as a unique model entity for immunological studies.

The outgrowth of MSI-H CRCs despite the presence of a dense lymphocytic infiltration suggests that several mechanisms interfere with the efficiency of the host's immune response *in vivo*. For example, immune evasion mechanisms such as impairment or loss of the human leukocyte antigen class I-mediated antigen presentation (Bicknell *et al*, 1996; Cabrera *et al*, 2003; Kloor *et al*, 2005) or the expression of Fas ligand (Okada *et al*, 2000; Michael-Robinson *et al*, 2003) are frequent in MSI-H CRCs.

In the recent past, regulatory T (Treg) cells and their role in the suppression of antitumoural immune responses have gained increasing attention. Treg cells represent a heterogeneous group of T cells that are defined on the basis of their ability to control the activation and function of antigen-reactive T cells in the periphery, thereby preventing self-reactivity (reviewed in Knutson *et al*, 2007). Treg cells can suppress reactivity of tumour antigen-specific T cells in CRC patients (Clarke *et al*, 2006). The number of Treg cells in the peripheral blood and the tumour itself are increased in patients suffering from gastrointestinal malignancies including CRCs (Ichihara *et al*, 2003; Sasada *et al*, 2003; Kono *et al*, 2006; Loddenkemper *et al*, 2006; Ling *et al*, 2007). This suggests that Treg cells may modulate the anti-tumoural immune response in CRC patients.

To date, it is unknown whether the well-established difference between MSI-H and MSS CRCs concerning the infiltration with lymphocytes of the cytotoxic and activated phenotype is paralleled by a difference in Treg cell infiltration. Recently, a novel Treg cell marker, forkhead box P3 (FOXP3), has been described (Fontenot *et al*, 2003; Hori *et al*, 2003; Khattri *et al*, 2003). The expression of FOXP3 in T cells corresponds with immune regulatory function (Hori *et al*, 2003; Fontenot *et al*, 2005; Roncador *et al*, 2005), indicating that the number of FOXP3-positive tumour-infiltrating lymphocytes is representative of the number of potential immune suppressor T cells in the tumour microenvironment (reviewed in Banham *et al*, 2006). To determine potential differences between Treg cell infiltration between MSI-H and MSS CRCs, we analysed infiltration with FOXP3-positive cells in 70 primary CRC lesions tested for their MSI status.

## Materials and methods

### Tumour samples and MSI analysis

Colorectal cancer samples were collected in the Heidelberg Centre for Familial Colorectal Cancer as part of a prospective study funded by the German Cancer Aid (Deutsche Krebshilfe) that has been approved by the Institutional Ethics Committee of the University of Heidelberg. Tumours were typed for MSI using the standard NCI/ICG-HNPCC marker panel (Boland *et al*, 1998) and CAT25 as described earlier (Findeisen *et al*, 2005). For tumour staging, the UICC/AJCC TNM system was applied (American Joint Committee on Cancer, 1997). Tumours of the patients who had received neo-adjuvant chemotherapy and tumours with a mucinous histology or extensive necrotic areas were excluded from the study. A total number of 37 MSI-H CRCs and 33 MSS CRCs were analysed.

### Immunohistochemistry

Tissue sections (2 *μ*m) were prepared from formalin-fixed, paraffin-embedded material and mounted on aminopropylsilane-coated slides (SuperFrost, Menzel, Braunschweig, Germany). After deparaffinisation and rehydration, the slides were boiled in 10 mM citrate buffer (pH 6) for 15 min to retrieve the antigens. Subsequently, the slides were allowed to cool for 30 min.

For the detection of CD3, CD8 and FOXP3 antigens, immunohistochemistry using ABC method was applied. The endogenous peroxidase activity was blocked by incubation with 0.6% H_2_O_2_ in methanol for 20 min. The sections were blocked with 10% normal horse serum (Vectastain Elite ABC kit, Vector, Burlingame, USA). For immunostaining, mouse monoclonal antibodies specifically recognising CD3 (1 : 50 dilution, clone PS1, Acris, Heford, Germany), CD8 (1 : 40 dilution, clone 4B11, Novocastra, Newcastle, UK) and FOXP3 (1 : 50 dilution, clone 236A/E7, eBioscience, San Diego, USA) were applied as primary antibodies at 4°C overnight. The slides were incubated with a biotinylated secondary antibody (1 : 50 dilution, horse anti-mouse IgG, Vectastain Elite ABC kit, Vector) for 30 min at room temperature and AB reagent was applied according to the manufacturer's instructions (Vectastain Elite ABC kit). The antigen detection was performed by a colour reaction with 3,3-diaminobenzidine (DAB+ chromogen, DakoCytomation, Glostrup, Denmark). The sections were counterstained with haematoxylin (AppliChem, Germany) and mounted with Aquatex (Merck).

For immunofluorescence staining, slides were co-incubated for 2 h at room temperature with the following monoclonal antibodies: mouse anti-CD3 (1 : 50 dilution, clone PS1, Acris) and rat anti-FOXP3 (1 : 100 dilution, clone PCH101, eBioscience) for CD3/FOXP3 double staining, mouse anti-CD8 (1 : 40 dilution, clone 4B11, Novocastra) and rat anti-FOXP3 (1 : 100 dilution, clone PCH101, eBioscience) for CD8/FOXP3 double staining. Secondary antibodies (goat anti-mouse IgG labelled with Alexa Fluor 488 and donkey anti-rat IgG labelled with Alexa Fluor 594; both from Molecular Probes, Eugene, OR, USA) were applied in a 1 : 50 dilution for 1 h at room temperature. Slides were counterstained with 4′,6-diamidino-2-phenylindol (2 *μ*g ml, Roche, Germany) and mounted with 9.6% Mowiol 4-88 (Roth, Karlsruhe, Germany) in Tris glycerol containing 0.1% DABCO (Roth). Negative controls were prepared by omitting the primary antibodies. In addition, incubation of rat primary antibodies with anti-mouse secondary antibody and vice versa was performed to exclude cross-reactivity of the secondary antibodies.

### Microscopic evaluation

Evaluation of the immunohistological stains was performed without knowledge of the MSI status of the tumour or clinical data. For counting and documentation of tumour-infiltrating lymphocytes, five representative fields (*i*=1 to *i*=5) of the tumour were chosen from each slide, and the stained cells were counted by means of a 10 × 10 ocular grid at × 200 magnification (observed area 0.25 mm^2^) using a Leica DMRBE microscope (Leica, Solms, Germany). Each field was subdivided into an epithelial and a stromal compartment, and for both compartments, cellular infiltration (*n*_*i*_) and the compartment's area (number of grid elements, *a*_*i*_) were recorded separately. The relative numbers of stained cells per 0.25 mm^2^ (*n*) were calculated for tumour epithelium and stroma using the following formula: 
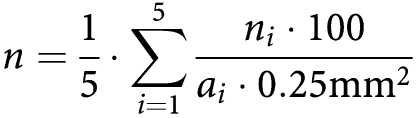
 Pictures were taken using a Leica DMRBE microscope (Leica) and a digital camera DFC480 (Leica), or scanned using a Hamamatsu NanoZoomer Digital Pathology system (Hamamatsu, Hamamatsu City, Japan). Immunofluorescence images were calculated presenting each fluorochrome signal with an artificial color: Alexa Fluor 488, green (CD3 and CD8, respectively), Alexa Fluor 594, red (FOXP3), and DAPI, blue (cell nuclei).

### Statistical analysis

The correlation between the numbers of FOXP3-positive, CD3-positive and CD8-positive cells infiltrating the tumour compartments was estimated using Spearman's rank correlation coefficient. The exact Mann–Whitney test was used for pairwise comparisons of the location of distributions of quantitative variables. Fisher's exact test was applied for pairwise comparisons of categorical variables. Two-sided tests were used for the pairwise comparisons. A univariate linear regression analysis was used to relate the numbers of FOXP3-positive cells to clinical variables. A result with a *P*-value lesser than 0.05 was always considered statistically significant. All statistical analyses were performed using R (R Development Core Team, 2007), version 2.6.1 together with the R package exactRankTests, version 0.8-16.

## Results

To compare the infiltration with FOXP3-positive cells in CRCs with different MSI status, we analysed 37 MSI-H CRCs and 33 MSS CRCs. At diagnosis, the age of patients with MSI-H CRCs was similar compared with the age of patients with MSS CRCs (median age 51 *vs* 49 years, respectively; *P*=0.92). Localisation in the proximal colon was more frequent in MSI-H CRCs than in MSS CRCs (*P*<0.001). There were no significant differences between the MSI-H and MSS groups concerning TNM or UICC stage distribution. Patient's characteristics are summarised in Table 1.

**Figure 1:**
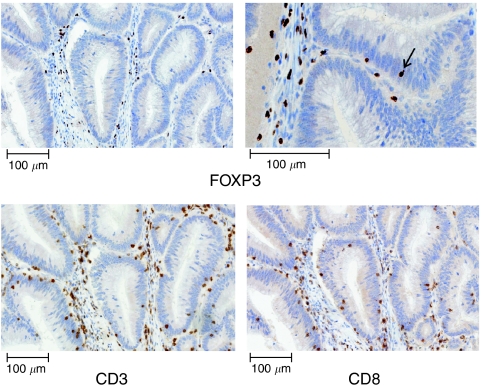
Representative immunohistochemical stainings with antibodies specific for FOXP3 (upper panel), CD3 (lower left panel) and CD8 (lower right panel). Detailed view of FOXP3 staining (upper right) shows the presence of FOXP3-positive cells infiltrating the tumour epithelium (arrow).

Infiltration of the tumour with FOXP3-positive cells was observed in all CRC specimens. In all tumours, the number of infiltrating FOXP3-positive cells was higher in the tumour stroma than in the epithelium. In addition to the staining for FOXP3, all tumours were stained with antibodies against CD3 and CD8 to assess the overall infiltration with CD3-positive T cells and with CD8-positive T cells. As observed for the FOXP3 stain, all tumours showed infiltration with CD3-positive and CD8-positive lymphocytes, which was higher in the tumour stroma than in the epithelium. The median values for the intraepithelial and stromal infiltration of MSI-H and MSS CRCs with FOXP3-positive, CD3-positive and CD8-positive lymphocytes are summarised in Table 2. The exemplary staining results are displayed in Figure 1.

**Figure 2:**
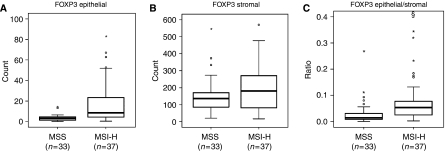
(**A**) Epithelial counts of FOXP3-positive cells in MSS and MSI-H colorectal cancers. (**B**) Stromal counts of FOXP3-positive cells in MSS and MSI-H colorectal cancers. (**C**) The ratio of epithelial-to-stromal FOXP3-positive cell counts in MSS and MSI-H colorectal cancers. *Y* axis was truncated at 0.4, § represents one extreme value at 0.7. Open circles represent outliers (above 1.5 interquartile ranges), ^*^ represent extreme values (above 3 interquartile ranges).

The comparison of MSI-H and MSS CRCs revealed a higher infiltration of MSI-H CRCs with FOXP3-positive cells. High-level microsatellite instability CRCs showed a trend towards a higher infiltration with FOXP3-positive cells in the tumour stroma (median: 181.5 cells per 0.25 mm^2^ in MSI-H *vs* 137.1 cells per 0.25 mm^2^ in MSS, *P*=0.06) and a significantly higher intraepithelial infiltration with FOXP3-positive Treg cells (median: 8.5 cells per 0.25 mm^2^ in MSI-H *vs* 3.1 cells per 0.25 mm^2^ in MSS, *P*<0.001). In addition, the ratio between intraepithelial and stromal FOXP3-positive cells was significantly higher in the MSI-H CRC group (0.05 in MSI-H *vs* 0.01 in MSS, *P*<0.001), indicating that in MSI-H CRC, a larger proportion of the FOXP3-positive cells was located in the epithelial compartment of the tumour. A graphical display of FOXP3-positive cell counts is shown in Figure 2. In accordance with previously published studies, we found higher numbers of intraepithelial CD3-positive T cells (median: 60.8 cells per 0.25 mm^2^ in MSI-H *vs* 14.1 cells per 0.25 mm^2^ in MSS, *P*<0.001) and CD8-positive T cells (median: 32.5 cells per 0.25 mm^2^ in MSI-H *vs* 6.3 cells per 0.25 mm^2^ in MSS, *P*<0.001) in MSI-H compared with MSS CRCs. In addition, the numbers of CD3-positive T cells (370.8 cells per 0.25 mm^2^ in MSI-H *vs* 320.1 cells per 0.25 mm^2^ in MSS, *P*=0.06) and CD8-positive cells (107.4 cells per 0.25 mm^2^ in MSI-H *vs* 47.2 cells per 0.25 mm^2^ in MSS *P*=0.009) infiltrating the tumour stroma were higher in MSI-H CRCs. The comparison of FOXP3-positive with CD8-positive cell counts revealed a positive correlation between the two markers (Spearman's rank correlation coefficient *ρ*=0.60, 95% confidence interval: 0.43–0.73).

In addition, we evaluated the association of clinicopathological variables with the intratumoural FOXP3-positive cell infiltration. We could not identify a statistically significant influence of age or gender on the overall infiltration of CRC with FOXP3-positive cells, yet a younger age was associated with a lower number of FOXP3-positive cells in the stroma and a higher ratio of epithelial-to-stromal infiltration with FOXP3-positive cells (*P*=0.07 and *P*=0.08, respectively). The infiltration with FOXP3-positive cells did not differ significantly between the local tumour stages (T1–T4). In CRC with nodal metastasis (N1/2), the number of stromal FOXP3-positive cells was significantly lower than in CRC without nodal metastasis (N0) (median: 136.9 cells per 0.25 mm^2^ in N1/2 *vs* 173.6 cells per 0.25 mm^2^ in N0, *P*=0.02), whereas the number of intraepithelial FOXP3-positive cells was not different between both groups (median: 5.4 cells per 0.25 mm^2^ in N1/2 *vs* 4.4 cells per 0.25 mm^2^ in N0, *P*=0.92). Similarly, tumours that had developed metastases in distant organs (M1) also showed a lower stromal infiltration with FOXP3-positive cells (median: 75.7 cells per 0.25 mm^2^ in M1 *vs* 171.6 cells per 0.25 mm^2^ in M0, *P*=0.01) compared with tumours without distant metastasis (M0), whereas the intraepithelial infiltration was not different (median: 5.1 cells per 0.25 mm^2^ in M1 *vs* 4.5 cells per 0.25 mm^2^ in M0, *P*=0.72).

**Figure 3:**
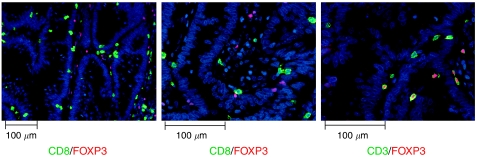
Representative immunofluorescence stainings. Double stainings for CD8 (Alexa Fluor 488, green) and FOXP3 (Alexa Fluor 594, red) in the left panel and centre panel, respectively. Double staining for CD3 (Alexa Fluor 488, green) and FOXP3 (Alexa Fluor 594, red) in the right panel.

Recent studies indicated that FOXP3 may be expressed transiently in CD8-positive effector T cells upon activation (Shevach, 2006; Baron *et al*, 2007). To further characterize the phenotype of FOXP3-positive cells detected in CRC lesions, immunofluorescence double staining was performed using antibodies specific for CD8/FOXP3, and CD3/FOXP3 as a control. Five CRC lesions showing high numbers of intraepithelial FOXP3 cells were selected for the analysis. Immunofluorescence stainings showed that all detectable FOXP3-positive cells were negative for CD8. In contrast, nuclear FOXP3 signals were regularly accompanied by membrane-bound CD3 staining. Exemplary images of immunofluorescence analysis are shown in Figure 3.

## Discussion

The role of Treg cells as suppressors of the host's antitumoural immune response has gained considerable interest in the recent past. The establishment of FOXP3 as a marker for Treg cells allowed for a phenotypic characterisation of the otherwise functionally defined group of Treg cells. Recent studies indicate that FOXP3 expression is not necessarily linked to a regulatory or suppressor phenotype in T cells, for example transient FOXP3 expression has been reported in activated CD8-positive effector T cells (Shevach, 2006; Baron *et al*, 2007). Therefore, it may be hypothesised that FOXP3 expression detected in intraepithelial lymphocytes might be ascribed to locally activated CD8+ cytotoxic T cells, particularly in MSI-H CRCs that are characterized by a high density of CD8-positive tumour-infiltrating T cells. Immunofluorescence double stainings for CD8/FOXP3 and CD3/FOXP3 as a control showed that no cells simultaneously expressing CD8 and FOXP3 were detectable in these lesions. Together with reports from the literature that tumour-infiltrating CD4-positive T cells can be detected in MSI-H as well as in MSS CRC stroma and at lower number also in the CRC epithelium (Dolcetti *et al*, 1999), these data show that the vast majority of FOXP3-positive T cells infiltrating CRC stroma and epithelium represent CD4-positive cells and not activated CD8-positive effector cells.

Initial studies on CRC specimens reported an elevated number of Treg cells in CRC compared with healthy colonic mucosa (Loddenkemper *et al*, 2006; Ling *et al*, 2007). However, Treg cell numbers varied between the tumour specimens; and there is little information about the influence of clinicopathological characteristics of the tumour on intratumoural Treg cell density. It is known from the literature that the number of tumour-infiltrating lymphocytes with a cytotoxic potential in CRC depends on the MSI status of the tumour. In analogy, one may hypothesise that MSI status may also have an influence on the density of tumour-infiltrating Treg cells.

Although a recent study has examined FOXP3 transcript levels in a series of CRCs that had been typed for MSI status (Le Gouvello *et al*, 2008), this study is the first to systematically analyse the infiltration with FOXP3-positive cells in CRC in dependence of MSI status. Although Le Gouvello *et al* (2008) observed higher expression levels of FOXP3 mRNA in MSS CRCs compared with MSI-H CRCs, this study detected an enhanced infiltration of FOXP3-postive cells in MSI-H CRCs by immunohistochemistry. This discrepancy might be attributed to the different methodology applied in the studies, and might in part reflect the notion that FOXP3 transcript may be present without the presence of FOXP3 protein (Yamamoto *et al*, 2008). In addition, the analysis of mRNA levels does not allow for the attribution of FOXP3 expression to specific cell types present in the tumour and may be influenced, for example, by tumour cells expressing FOXP3 mRNA (Hinz *et al*, 2007).

For our analysis, we chose to differentiate between intraepithelial and stromal infiltration, considering an earlier observation that the prognostic significance of lymphocyte infiltration was mainly related to the number of intraepithelial lymphocytes (Naito *et al*, 1998; Sato *et al*, 2005). In our collection, we observed a significantly higher number of FOXP3-positive Treg cells in the stroma compared with the epithelial compartment of the tumour, which is in accordance with the results reported by Loddenkemper *et al* (2006). However, in contrast to the previously reported absence of Treg cells in tumour epithelium (Ling *et al*, 2007), intraepithelial FOXP3-positive cells, although sparse in some tumours, were detected in all but one of the analysed lesions.

The comparison of MSI-H and MSS CRCs revealed a significantly higher number of intraepithelial FOXP3-positive lymphocytes in MSI-H compared with MSS CRCs and a trend towards a higher infiltration with these cells in the tumour stroma of MSI-H CRCs. Also in MSI-H CRCs, a higher proportion of the tumour-infiltrating FOXP3-positive cells were located in the epithelium, as indicated by a higher ratio of epithelial-to-stromal infiltration in the MSI-H group. This indicates that the previously known difference between MSI-H and MSS CRCs concerning overall lymphocytic infiltration as well as the infiltration with CD3-positive and CD8-positive T cells also extends to a differential infiltration with FOXP3-positive Treg cells. Moreover, a close association of FOXP3-positive cell counts with numbers of CD8-positive cells was shown (Spearman's rank correlation).

In addition, we looked at several other clinicopathological parameters that may be associated with Treg cell counts, although we concede that the number of tumours included in the study limits the power of the statistical analysis. No significant difference in infiltration with FOXP3-positive cells could be detected between tumours of different local tumour stages (T1–T4), potentially reflecting the limited number of tumour samples in each group. The density of FOXP3-positive cells in tumour stroma was significantly higher in unmetastasised CRCs compared with tumours with lymph node or distant metastases. These data are in accordance with the findings of Loddenkemper *et al*, who reported a significantly higher Treg infiltration in limited than in metastatic CRC that was mainly caused by Treg infiltrating the tumour stroma. In contrast, no difference in intraepithelial infiltration was observed. Two studies (Atreya *et al*, 2007; Ling *et al*, 2007) failed to find an association of intratumoural FOXP3-positive Treg cells or FOXP3 mRNA expression with the presence or absence of metastases. At present, the significance of Treg cell infiltration for the progression of CRC remains unclear and warrants further investigation. Similarly, the association of Treg cell counts with overall or disease-free survival was not feasible in our collection of patients. Concerning the mechanism underlying the increased numbers of tumour-infiltrating FOXP3-positive cells observed in CRCs of the MSI-H group, it is interesting to note that dendritic cells can expand Treg populations (Nagorsen *et al*, 2007), thus potentially explaining an increased frequency of FOXP3-positive cells in MSI-H CRCs as a consequence of a pronounced immune response observed in these lesions. The mechanisms leading to the observation of enhanced FOXP3-positive cell counts in MSI-H CRCs and its clinical significance need to be addressed in future studies.

In summary, this is the first study systematically examining the association of mismatch repair deficiency and infiltration with FOXP3-positive cells. Our results suggest that the density of Treg cells infiltrating CRCs is significantly higher in MSI-H compared with MSS CRCs, thus paralleling the enhanced number of CD8-positive cells in these tumours. A dense infiltration of MSI-H CRCs with FOXP3-positive cells may play a role in local MSI-H tumour growth in the presence of potentially cytotoxic T cells in the local tumour environment.

## Figures and Tables

**Table 1:** Characteristics of CRC patients

		**MSA status**		
	**Total**	**MSI-H**	**MSS**	***P*-value^a^**
Number of patients	*n*=70	*n*=37 (52.9)	*n*=33 (47.1)	
Age (years; median, range)	50 (29–82)^b^	51 (29–82)	49 (29–73)	0.92
				
*Gender*				0.16
Male	36 (51.4)	16 (43.2)	20 (60.6)	
Female	34 (48.6)	21 (56.8)	13 (39.4)	
				
*Localisation*				<0.001
Proximal	31 (44.3)	24 (64.9)	7 (21.2)	
Distal	28 (40.0)	6 (16.2)	22 (66.7)	
NA	11 (15.7)	7 (18.9)	4 (12.1)	
				
*Primary tumour*				0.37
T1	4 (5.7)	2 (5.4)	2 (6.1)	
T2	13 (18.6)	4 (10.8)	9 (27.3)	
T3	41 (58.6)	24 (64.9)	17 (51.5)	
T4	8 (11.4)	4 (10.8)	4 (12.1)	
Tx	4 (5.7)	3 (8.1)	1 (3.0)	
				
*Lymph node status*				0.94
N0	32 (45.7)	17 (46.0)	15 (45.5)	
N1/N2	27 (38.6)	16 (43.2)	11 (33.3)	
Nx	11 (15.7)	4 (10.8)	7 (21.2)	
				
*Distant metastasis*				0.49
M0	42 (60)	27 (73)	15 (45.5)	
M1	8 (11.4)	4 (10.8)	4 (12.1)	
Mx	20 (28.6)	6 (16.2)	14 (42.4)	
				
*Stage of disease (UICC)*				0.64
I	10 (14.3)	5 (13.5)	5 (15.2)	
II	17 (24.3)	11 (29.7)	6 (18.2)	
III	14 (20.0)	10 (27.0)	4 (12.1)	
IV	8 (11.4)	4 (10.8)	4 (12.1)	
NA	21 (30.0)	7 (18.9)	14 (42.4)	

NA=not analysable.

a Fisher's exact test for categorical data and the Mann–Whitney test for age.

b Numbers in parentheses indicate percentage values if not otherwise indicated.

**Table 2:** Intratumoural infiltration with positively stained cells

	**Intraepithelial**	**Stromal**	**Intraepithelial/stromal**
(a) *Infiltration with FOXP3-positive cells (median number of cells per 0.25 mm*^*2*^*)*			
MSI-H CRC	8.5	181.5	0.05
MSS CRC	3.1	137.1	0.01
			
*P*-value	<0.001	0.06	<0.001
			
(b) *Infiltration with CD3-positive cells (median number of cells per 0.25 mm*^*2*^*)*			
MSI-H CRC	60.8	370.8	0.18
MSS CRC	14.1	320.1	0.05
			
*P*-value	<0.001	0.06	<0.001
			
(c) *Infiltration with CD8-positive cells (median number of cells per 0.25 mm*^*2*^*)*			
MSI-H CRC	32.5	107.4	0.30
MSS CRC	6.3	47.7	0.11
			
*P*-value	<0.001	0.009	<0.001

All *P*-values were computed using the exact Mann–Whitney test.
